# PERCEIVED BENEFITS OF TREATMENTS OF ALZHEIMER’S DISEASE AND RELATED DEMENTIAS FOR COST-EFFECTIVENESS ESTIMATES

**DOI:** 10.1093/geroni/igae098.3683

**Published:** 2024-12-31

**Authors:** Faika Zanjani, Madeline McIntyre, Tracey Gendron, Eline van den Broek-Altenburg

**Affiliations:** Virginia Commonwealth University, Richmond, Virginia, United States; VCU, Richmond, Virginia, United States; Virginia Commonwealth University, Richmond, Virginia, United States; University of Vermont, Burlington, Vermont, United States

## Abstract

**Objective:**

This study aims to identify important non-Quality-adjusted life years (QALYs) benefits of novel Alzheimer’s Disease and Related Dementia (AD/ADRD) treatments. Cost-effectiveness analysis of AD/ADRD is mostly based on QALYs gained. However, other benefits in the International Society for Pharmacoeconomics and Outcomes Research value flower, such as severity of disease, hope, and option value are not included. Which of these benefits are most important for AD/ADRD is unknown.

**Methods:**

Twelve semi-structured focus groups were conducted with at-risk individuals (60%) and caregivers (40%). Study protocol/interview guide was designed to target AD/ADRD knowledge and attitudes. Focus groups were recorded. transcribed, and analyzed thematically using ATLAS.ti and two coders.

**Results:**

There was a lack of knowledge about both the condition and treatments. When considering treatment, cost and side effects were important, but impact on family dynamics was also critical. Participants lack trust in advertising, confused by the healthcare system, feel primary care providers are undertrained and lack time, and specialists are unavailable; the whole system lacks coordination, fails to communicate effectively, and provides conflicting information. Participants indicated AD/ADRD is associated with grief, sadness, fear, frustration, and hopelessness. They also expressed feeling alone and confused. Caregiving at a distance presents a particular challenge. Conclusions: Omitted elements of hope and option value from the value flower are important for AD/ADRD, suggesting that current cost-effectiveness estimates may systematically undervalue important components of treatments. Yet even with new medications, access challenges may undermine potential value by creating systematic challenges in receiving diagnoses and identifying treatment alternatives.

